# Genetic variation in macrophage-restricted FOLR2 is associated with neurodevelopmental and reproductive immune phenotypes

**DOI:** 10.3389/fimmu.2026.1804188

**Published:** 2026-09-10

**Authors:** Elizabeth A. Jasper, James Jaworski, Yuan Luo, Bahram Namjou Khales, Atlas Khan, Digna R. Velez Edwards, David M. Aronoff

**Affiliations:** 1 Department of Obstetrics and Gynecology, Division of Quantitative and Clinical Sciences, Vanderbilt University Medical Center, Nashville, TN, United States; 2 Department of Biomedical Informatics, Vanderbilt University Medical Center, Nashville, TN, United States; 3 Division of Epidemiology, Department of Medicine, Vanderbilt University Medical Center, Nashville, TN, United States; 4 Department of Preventive Medicine, Feinberg School of Medicine, Northwestern University, Chicago, IL, United States; 5 Department of Pediatrics, Cincinnati Children’s Hospital Medical Center, Cincinnati, OH, United States; 6 Division of Nephrology, Department of Medicine, Columbia University, New York City, NY, United States; 7 Division of Infectious Diseases, Department of Medicine, Indiana University School of Medicine, Indianapolis, IN, United States; 8 Indiana University Health, Indianapolis, IN, United States

**Keywords:** folate, FOLR2, hypertension, macrophage, neurodevelopmental disorders, placenta, pregnancy

## Abstract

**Introduction:**

Folate receptor beta (FRβ), encoded by *FOLR2*, is a cell surface receptor with restricted expression in monocytes and macrophages and is highly expressed at the maternal-fetal interface, yet its genetic contributions to human disease remain unexplored.

**Methods:**

Given the importance of placental macrophages to host defense, including against viral infections, we performed a gene-based phenome-wide association study (PheWAS) using three classes of *FOLR2* variation—rare functional and regulatory, rare functional coding, and protein-altering variants—across 170,889 individuals from two large, independent electronic health record–linked biobanks (BioVU and eMERGE).

**Results:**

In ancestry-specific and cross-ancestry meta-analyses, we identified significant associations between *FOLR2* variation and phenotypes including pervasive developmental disorders such as attention-deficit hyperactivity disorder, genitourinary infections in pregnancy, and tension headache (P < 3.95 × 10^-5^). Additional suggestive associations included dyspareunia and ocular inflammation.

**Discussion:**

These findings uncover previously unrecognized links between *FOLR2* variation and human phenotypes, providing genetic evidence that folate receptor beta may influence neurodevelopmental and immune-mediated traits. Our study highlights *FOLR2* as a candidate gene of interest in the biology of macrophage-related disease and reproductive immunology.

## Introduction

Folates (vitamin B9) are essential micronutrients that support nucleic acid and amino acid synthesis, making them critical during periods of rapid cell division such as pregnancy (1, 2). Folate deficiency during pregnancy causes fetal neural tube defects—including anencephaly, encephalocele, and spina bifida—and periconceptional folic acid supplementation substantially reduces their incidence (3, 4). Because cellular folate uptake depends critically on folate receptors and transporters, the biology and genetics of these proteins have direct relevance to human health and disease.

As folates are not synthesized by eukaryotic cells, they are delivered into cells through transporters such as the ubiquitously-expressed reduced folate carrier [RFC; encoded by the *SLC19A1* gene (5)], or the proton-coupled folate transporter [PCFT, *SLC46A1* gene (6)], which is involved in dietary folate uptake from the intestine. In addition, folates can bind to a family of cell membrane, glycosyl-phosphatidylinositol (GPI)-anchored folate receptors (FRs) (7). Contrary to the common folate transporters RFC and PCFT, these FRs, including FRα (encoded by the *FOLR1* gene), FRβ (encoded by *FOLR2*), and FRδ (encoded by *FOLR4*, also known as Juno) exhibit highly cell type-specific expression. The exact functions of these FRs are unclear but there are studies showing that FRα and FRβ internalize extracellular folates through a process of endocytosis and receptor recycling back to the cell surface (1, 8, 9). Endocytosis, however, is not as efficient at folate importation as is shuttling folate via the RFC transporter (10). FRδ (*aka*, Juno) is GPI-anchored and expressed largely on oocytes, however it is primarily involved in the process of fertilization and does not appear to bind folates (11). A fourth FR, FRγ (encoded by *FOLR3*), lacks a GPI tail, is secreted from myeloid cells, and is also not thought to be involved in folate transport (12).

Expression of the FRβ-encoding *FOLR2* gene is remarkably specific for monocytes and macrophages (1, 13). The placenta is the human organ with the highest expression of *FOLR2* (14–16), due to strong expression by placental macrophages (both fetal and maternal) (17–21). How this receptor influences cell behavior is not defined (22) and these cells also express RFC and PCFT to foster folate importation (23, 24). We recently used CRISPR/Cas9 technology to delete the *FOLR2* gene from the macrophage-like Tohoku Hospital Pediatrics-1 (THP-1) cell line and found FRβ influences DNA methylation, gene expression, and the inflammatory response to immune stimulation, through folate-independent mechanisms (25).

Given the importance of macrophages to host defense, the specific association of *FOLR2* expression with these cells, and *in vitro* data suggesting a role for FRβ in modulating macrophage biology, we speculated that this protein might play a role in governing innate immunity and tissue inflammation. Because expression is enriched at the maternal-fetal interface we also hypothesized that pregnancy outcomes might be influenced by FRβ, whatever its exact function. Placental macrophages have been implicated in the response to viral infections, including Zika virus (26), SARS-CoV-2 (27), CMV (28), and HIV (28).

Polymorphisms in the *FOLR2* gene have not been systematically studied for their potential associations with human diseases, despite the growing recognition of folate metabolism and transport in immune-mediated disorders. Genome-wide association studies (GWAS) and candidate gene studies have identified roles for folate-related genes in neural tube defects, cardiovascular diseases, and certain cancers, but these studies have largely focused on FRα or folate metabolism enzymes such as *MTHFR* (29–32). By contrast, the role of *FOLR2* in human health and disease has received minimal attention, creating a significant knowledge gap in our understanding of FRβ-mediated biological processes.

To address this gap, we performed a phenome-wide association study (PheWAS) to systematically evaluate associations between genetic polymorphisms in *FOLR2* and a wide range of human phenotypes. PheWAS is a hypothesis-generating approach that leverages large-scale biobank datasets to identify potential links between genetic variants and diverse clinical phenotypes, enabling an unbiased exploration of gene-disease relationships (33). By applying PheWAS to the *FOLR2* gene, we aimed to uncover novel insights into the biological roles of FRβ, particularly its contributions to innate immunity and its potential involvement in inflammatory and immune-related diseases. This study represents a critical step toward understanding the underexplored functions of folate receptor beta in human immunology.

## Materials and methods

### Study populations

Our study populations were obtained from two sources: BioVU and the Electronic Medical Record and Genomics (eMERGE) Network (34, 35). BioVU is a biorepository linking DNA samples to deidentified electronic health records (EHR) for patients receiving care at Vanderbilt University Medical Center (VUMC) (34). EMERGE is a consortium of several EHR-linked biorepositories from across the United States. It was formed with the goal of developing approaches for the use of the EHR in genomic research. Sites contributing data to eMERGE include Group Health/University of Washington, Marshfield Clinic, Mayo Clinic, Northwestern University, Vanderbilt University, Children’s Hospital of Philadelphia (CHOP), Boston Children’s Hospital (BCH), Cincinnati Children’s Hospital Medical Center (CCHMC), Geisinger Health System, Mount Sinai School of Medicine, Harvard University and Columbia University (35). While VUMC is an eMERGE site, the data it contributes to eMERGE is independent and does not overlap with data in BioVU. Detailed description of the database and how it was maintained has been published elsewhere (34, 35). Individuals were eligible for inclusion in the study if they had existing genotype data and were non-Hispanic European or African genetic ancestry. Genetic ancestry for BioVU samples was inferred using ADMIXTURE version 1.3.0 with reference populations from the 1000 Genomes project. For the eMERGE dataset principal component analysis was used to quantify population stratification and account for genetic ancestry variation. Other ancestries were excluded due to small sample sizes. This study was deemed non-human subjects research and approved by the VUMC Institutional Review Board.

### Genotyping

The BioVU DNA samples were genotyped using the custom Illumina Multi-Ethnic Genotyping Array (Mega-ex; Illumina Inc., San Diego, CA, USA) and subsequently imputed using the Michigan Imputation Server on the TOPMED reference panel (Version.r2.2020) (36, 37). Genotyping methods for samples from eMERGE are previously described (38). Using the Michigan Imputation Server, genotyped eMERGE samples were imputed to the Haplotype Reference Consortium (HRC) panel v1.1 (36, 39). Previously described quality control procedures, such as strand orientation, were performed for eMERGE prior to merging genotype data from each contributing site. Standard sample and marker quality control procedures were performed on both datasets prior to analyses. Sample quality control consisted of removing individuals who revoked consent, had compromised samples, genotype call rates below 98%, or failed sex concordance checks. Sample relatedness was also evaluated, and relatives were removed. Imputed variants were hard called with an info score above 0.9. Variants were excluded if they had call rates of less than 98% or deviated from Hardy Weinberg Equilibrium. To meta-analyze results accurately, the eMERGE dataset was lifted over to build Hg38 to match the BioVU dataset. The variants were harmonized across ancestries for the eMERGE and BioVU datasets, matching strand and ref/alt alleles.

### *FOLR2* genetic variants and analytical models

After quality control, we further limited variants in the analyses to focus on those in the *FOLR2* gene. The Ensembl Homo sapiens gene map (GRCh38.p3) was used to define the genomic boundaries of the *FOLR2* gene, extended by an additional 100 bp (72,216,551-72,222,000) (40). Variants within the gene were extracted and annotated using the Ensembl Variant effect predictor. The following three variant analysis models, defined based on consequence and impact on the *FOLR2* gene, were used for the gene-based PheWAS:

1. Rare functional and regulatory: all rare variants (minor allele frequency <0.05) except those annotated as synonymous. This set includes modifier, moderate, and high-impact consequences and incorporates variants within the 100 bp gene boundary used for gene-based testing.
2. Rare functional coding: all rare variants with functional coding consequences, with some modifier variants included depending on their annotation category.
3. Protein-altering: only rare, moderate- and high-impact variants, includes missense, start-lost, stop-gain, frameshift, and splice acceptor variants.

The three gene-based models were nested, with the rare functional and regulatory model composed of the largest number of variants from the *FOLR2* gene, followed by the rare functional coding model. The protein-altering model was the most restrictive and contained the smallest number of variants (**Table 1**; **Supplementary Table 1**).

**Table 1:** Populations and models.

	BioVU		eMERGE	
	African ancestry	European ancestry	African ancestry	European ancestry
Total sample (N)	13621	60192	14895	82181
Sex, n (%)				
Females	8,481 (62.26)	33,637 (55.88)	10,364 (69.58)	43,057 (52.39)
Males	5,140 (37.74)	26,555 (44.12)	4,531 (30.42)	39,124 (47.61)
Age, mean (standard deviation)	47 (17.83)	57 (17.61)	42 (24.17)	57 (23.70)
FOLR2 models (variant number)				
Rare functional and regulatory model	137	175	29	46
Rare functional coding model	40	36	7	7
Protein-altering model	19	20	5	6

### Phenome-wide association analyses

Phecodes were assigned using the Phecode Map v1.2, which aggregates ICD-9 and ICD-10-CM billing codes into clinically meaningful phenotype groups (33). We then conducted PheWAS, evaluating associations of up to 1,856 clinical phenotypes (phecodes) and the separate three gene-based models (33). All analysis were performed using R version 3.6.0. Gene-based PheWAS tests were conducted using the SKAT R package (41). Ancestry-specific analysis were run using SKATBinary_Robust function with the SKAT-O option, which adaptively combines the kernel-based SKAT statistic and the burden statistic by optimizing over a grid of Rho values (0-1). The robust option was used to account for case and control sample size imbalance present for many phecodes in our study populations (41). In instances where the imbalance could not be fully corrected by standard SKAT-O, the robust method ensured valid inference by returning a corrected, variance-adjusted SKAT statistic. A separate burden test was also performed using the SKATBinary Robust option. Effect size estimates and directionality for the burden tests were obtained by regressing the variant burden score on the covariates. Burden odds ratios were generated for highlighted associations and are provided as complementary summaries of directionality (**Supplementary Table 7**); they are not equivalent to the effect size for SKAT/SKAT-O or MetaSKAT, which model heterogeneous variant effects. For both the SKAT-O and burden analyses, we used the default linear weighted kernel with weights.beta=c (1, 25) (the standard rare-variant Beta kernel), and missingness cutoff of 0.15. All analyses were adjusted for sex, age, and 10 principal components.

**Figure 1:**
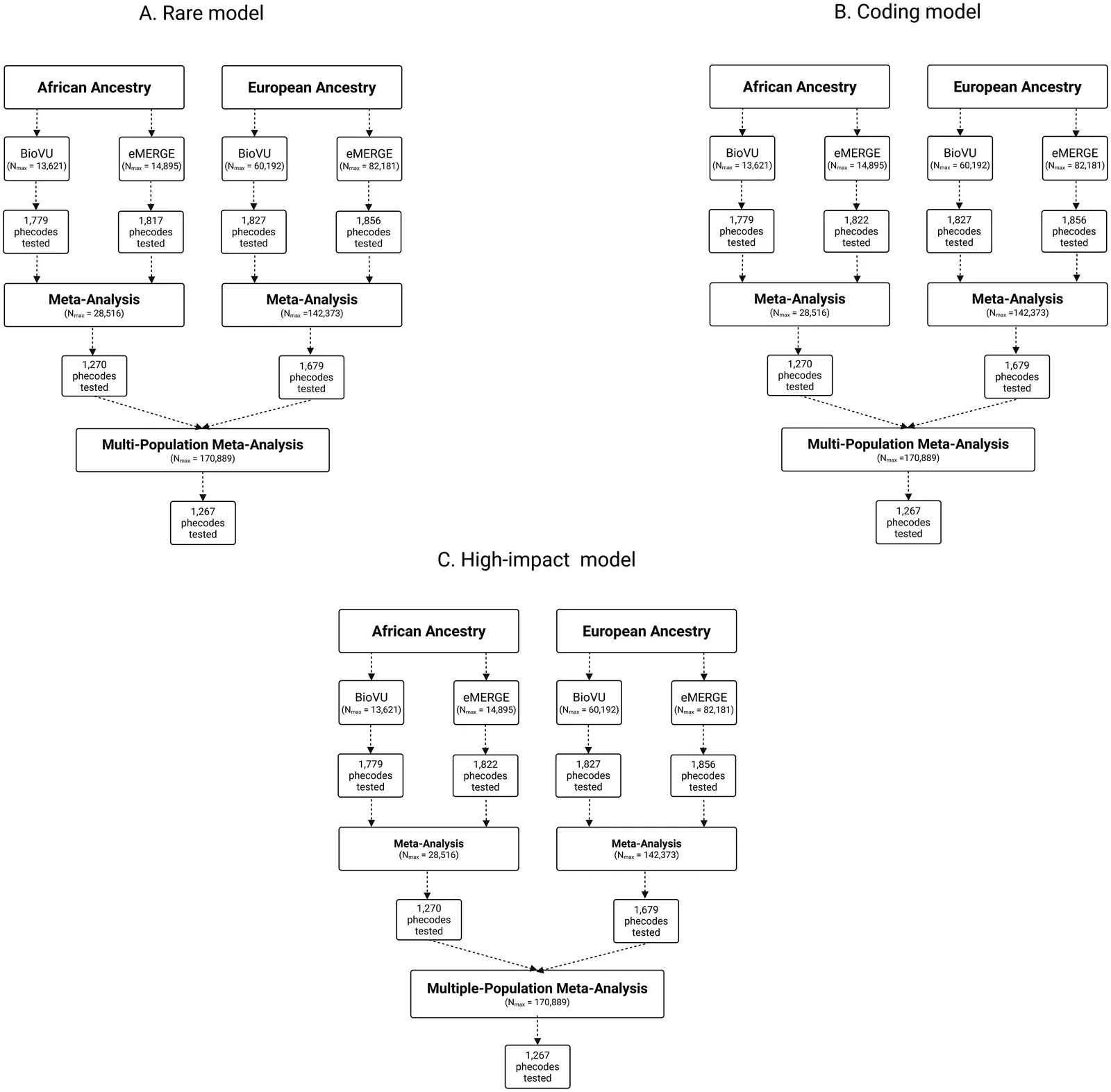
Analysis flow diagram. Analytic workflow for the three gene-based models: rare functional regulatory **(A)**, functional coding **(B)**, and protein-altering **(C)**. SKAT tests were performed independently in BioVU and eMERGE for African-ancestry and European-ancestry cohorts across all phecodes. Ancestry-specific meta-analyses were conducted, with N_max denoting the largest case-control count observed across phecodes. A final cross-ancestry meta-analysis combined results from both populations.

PheWAS were first performed separately for each ancestry and database (e.g., non-Hispanic African ancestry BioVU, non-Hispanic African ancestry eMERGE; **Figure 1**). The R package MetaSKAT was utilized to perform ancestry specific meta-analyses. MetaSKAT aligns variants by position and ID across ancestries, combines the score vectors and covariance matrices, and computes the SKAT test at the meta-analysis level. SKAT is well suited for rare-variant aggregation because it remains powerful when variant effects differ in direction or magnitude. We used the MetaSKAT_MSSD_All function with default parameters, including combined.weight=TRUE, weights.beta = c (1,25), method=“ davies” for p-value computation, r.corr = 0 (corresponding to classical SKAT model), is.separate=TRUE, and a missingness cutoff of 0.15. To be included in the meta-analysis, phecodes had to have suggestive significance in at least one of the populations and a minimum of 20 cases in each of the datasets being meta-analyzed. P-values of less than 0.05 were considered to have suggestive significance. Therefore, PheWAS in different populations tested different numbers of phecodes with different Bonferroni corrections determining significance. We also performed multi-ancestry meta-analysis using MetaSKAT, using only phecodes that had suggestive significance associated with any of the three gene-based models and in either ancestry. A Bonferroni correction based on the number of tests in each PheWAS was used to determine significance (roughly 3.93x10^-5^ for non-Hispanic African ancestry meta-analysis, 2.98x10^-5^ for non-Hispanic European ancestry meta-analysis, and 3.95x10^-5^ for multi-ancestry rare functional and regulatory, functional coding, and protein-altering model meta-analysis, respectively.

Given folates critical role in gestation, we performed sensitivity analyses for pregnancy complications. These analyses were limited to females with confirmed pregnancy history. Mirroring the overall approach, PheWAS were first performed separately for each ancestry and database for each of the three gene-based models prior to meta-analysis. Bonferroni correction was used to determine an adjusted level of significance (roughly 1.85x10^-3^ for African ancestry meta-analysis, 1.35x10^-3^ for European ancestry meta-analysis, and 1.85x10^-3^ for multi-ancestry meta-analysis for rare functional and regulatory, functional coding, and protein-altering models, respectively). Associations with *p*-values of less than 0.05 were also considered suggestive.

## Results

### Study population and gene-based models

This study included 170,889 individuals (**Table 1**). Roughly 43.2% (N = 73,813) of the total cohort was obtained from BioVU and the remaining 56.8% (N = 97,076) of the total study population was from eMERGE. In both databases and overall, approximately 20% of individuals were of African ancestry. In all databases and ancestries, there were more females than males. The African ancestry populations were, in general, younger than the European ancestry populations. Rare non-synonymous variants were more common in those of European ancestry. The number of functional coding and protein-altering *FOLR2* variants were roughly equal between ancestries with 5 high impact consequences represented in the models (**Supplementary Table 6**). For all models, more variants were available in the BioVU populations compared to the eMERGE populations (**Table 1**; **Supplementary Table 1**). In the analysis using only individuals with a history of pregnancy, a maximum of 13,985 individuals were included. Over half of the individuals were from eMERGE (53.60%). Approximately 27% of the pregnant cohort were of African ancestry.

### Dataset and ancestry-specific gene-based PheWAS

The gene-based PheWAS was first performed separately for the two ancestries and in the BioVU and eMERGE datasets. In BioVU, 1,779 phenotypes were tested for association with the rare functional and regulatory, functional coding, and protein-altering models for the African ancestry population, while 1,827 tests were performed in the European populations. There were no significant associations in any of the BioVU analyses after multiple testing correction (**Supplementary Tables 2; 3**). However, there were multiple suggestive associations, including infections of the genitourinary tract during pregnancy in both the functional coding variant (*p*-value: 9.40x10^-5^) and the protein-altering variant model (*p*-value: 3.83x10^-4^) in individuals of African ancestry, MRSA pneumonia in the African ancestry rare gene model (*p*-value: 3.30x10^-4^), and pervasive developmental disorders in the European ancestry functional coding (*p*-value: 9.60x10^-5^) and protein-altering (*p*-value: 9.03x10^-5^) variant models.

For the African ancestry population in eMERGE, 1,817 associations were tested in the rare functional and regulatory variant model and 1,822 in the functional coding and protein-altering variant models. There were 1,856 associations tested in the European population from eMERGE. After correction for multiple testing, there were no significant associations in any ancestry or gene-based model. Again, however, there were numerous suggestive associations, including vitamin D deficiency in the functional coding variant (*p*-value: 1.30x10^-3^) and protein-atlering (*p*-value: 8.91x10^-4^) gene model in those of African ancestry, and early onset of delivery (*p*-value: 2.69x10^-3^) in the protein-altering model in those with European ancestry (**Supplementary Tables 2, 3**).

### Ancestry-specific meta-analyses

We performed ancestry-specific gene-based PheWAS, combining ancestries from BioVU and eMERGE and limiting phenotypes to those that had at least 20 cases in both datasets. In the African ancestry meta-analyses, 1,270 phenotypes were tested for associations with the rare functional and regulatory, functional coding, and protein-altering variant models. A single outcome, inflammation of the eye, was associated with the rare functional and regulatory variant *FOLR2* model (*p*-value: 6.02x10^-6^). Eighty-four phenotypes displayed suggestive significance in the African ancestry rare functional and regulatory gene-based PheWAS, including nonallopathic lesions (not elsewhere classified), dyspareunia, and infection of genitourinary tract during pregnancy (**Supplementary Table 3**). Dyspareunia and genitourinary tract infections during pregnancy were the top and only phenotypes significantly associated with both the functional coding and protein-altering variant models in the African ancestry meta-analysis (**Table 2**). Roughly 50 phenotypes exhibited suggestive significance in both the functional coding (N = 52) and protein-altering (N = 47) models in the meta-analysis. The phenotypes that had suggestive significance in PheWAS using the functional coding model, including Barrett’s esophagus, asthma, mental disorders during/after pregnancy, and renal colic, generally also exhibited suggestive significance with the protein-altering *FOLR2* model (**Table 2**; **Supplementary Table 3**).

**Table 2:** Ancestry-specific meta-analysis.

			African ancestry												European ancestry											
			Rare functional and regulatory model				Functional coding variant model				Protein-altering variant model				Rare functional and regulatory model				Functional coding variant model				Protein-altering variant model			
Phecode	Description	Group	P-value	BioVU cases	eMERGE cases	Total	P-value	BioVU cases	eMERGE cases	Total	P-value	BioVU cases	eMERGE cases	Total	P-value	BioVU cases	eMERGE cases	Total	P-value	BioVU cases	eMERGE cases	Total	P-value	BioVU cases	eMERGE cases	Total
130	Spirochetal infection	Infectious disease													0.00056553	47	416	463	0.62049255	47	416	463	0.68558494	47	416	463
170.1	Bone cancer	Neoplasms													0.35702566	389	433	822	0.00030535	389	433	822	0.00028956	389	433	822
175	Acquired absence of breast	Neoplasms	0.00107836	109	48	157	0.21003447	109	48	157	0.19473558	109	48	157												
306.1	Mental disorders durring/after pregnancy	Mental disorders	0.05240545	105	29	134	0.00089831	105	29	134	0.0006588	105	29	134												
306.9	Tension headache	Mental disorders													8.6754E-05	66	836	902	0.1877972	66	836	902	0.2001439	66	836	902
313	Pervasive developmental disorders	Mental disorders													0.05429454	1613	3260	4873	**8.2711E-06**	1613	3260	4873	**8.4344E-06**	1613	3260	4873
313.1	Attention deficit hyperactivity disorder	Mental disorders													0.04402709	1104	2416	3520	8.7877E-05	1104	2416	3520	8.8249E-05	1104	2416	3520
371	Inflammation of the eye	Sense organs	**6.0202E-06**	393	604	997	0.78463739	394	604	998	0.66744217	394	604	998												
402	Elevated blood pressure reading without diagnosis of hypertension														0.00019809	321	819	1140	0.42517487	321	819	1140	0.53060312	321	819	1140
430.3	Subdural hemorrhage	Circulatory system													0.17273612	280	434	714	0.00013656	280	434	714	0.00013031	280	434	714
495	Asthma	Respiratory	0.00142226	1421	3503	4924	0.00067551	1425	3503	4928	0.00065451	1425	3503	4928												
535.2	Atrophic gastritis	Digestive					0.04830617	85	130	215					0.41439036	443	466	909	0.00032388	443	466	909	0.00035677	443	466	909
588.2	Secondary hyperparathyroidism (of renal origin)	Genitourinary													0.00014973	281	880	1161	0.1973044	281	880	1161	0.46429918	281	880	1161
625.1	Dyspareunia	Genitourinary	0.0003909	39	44	83	**3.5928E-06**	39	44	83	**3.8762E-06**	39	44	83												
647.1	Infections of genitourinary tract during pregnancy	Pregnancy complications	0.00055026	179	54	233	**3.8967E-06**	180	54	234	**1.562E-05**	180	54	234												
733.2	Cyst of bone	Musculoskeletal													0.00051745	65	141	206	0.78182312	65	141	206	0.6675259	65	141	206
736.6	Unequal leg length (acquired)	Musculoskeletal	0.0149855	20	34	54	0.00104852	20	34	54	0.000951	20	34	54												
769	Nonallopathic lesions NEC	Symptoms	8.3337E-05	21	24	45	0.75132236	21	24	45	0.62275471	21	24	45												

Bold font represents statistical singificance.

In the European meta-analysis, 1,679 phenotypes were tested for associations with the three models. Though no associations were statistically significant after correcting for multiple testing in the rare functional and regulatory variant PheWAS, there were 96 suggestive hits (**Table 2**; **Supplementary Table 4**). A single phenotype was significantly associated with both the functional coding and protein-altering models: pervasive developmental disorders (*p*-values < 8.45x10^-6^). One hundred and sixty-three phenotypes had suggestive significance in the coding model PheWAS, compared to 96 phenotypes in the high impact model PheWAS (**Table 2**; **Supplementary Table 4**). The majority of the phenotypes with suggestive significance in the functional coding gene-based model also had borderline significance in the protein-altering gene-based model.

### Multi-ancestry meta-analyses

**Figure 2:**
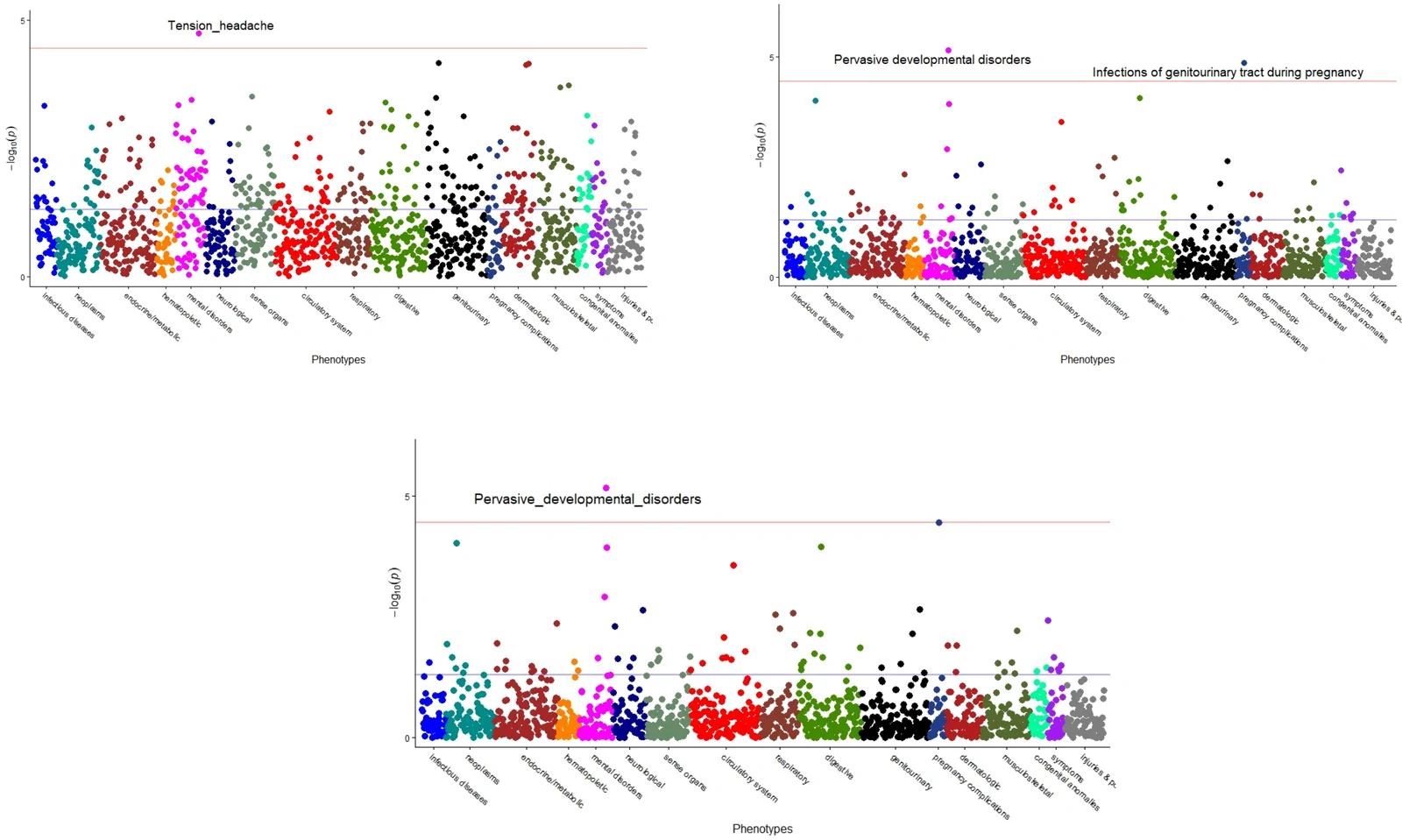
Manhattan plot of phenome wide association study results for the rare functional and regulatory **(A)**, rare functional coding **(B)**, and protein-altering **(C)** *FOLR2* gene-based models in the multi-ancestry meta-analyses.

When meta-analyzing across ancestries and databases, 1,267 phenotypes were examined for associations with models. Two phenotypes were significantly associated with the rare functional and regulatory variant model (**Table 3**): tension headache (*p*-value: 1.79x10^-5^) and elevated blood pressure reading without diagnosis (*p*-value: 2.72x10^-5^) (**Figure 2**). Over 300 phenotypes were marginally significant, most of which were mental, genitourinary, or sense organ disorders. Pervasive developmental disorders and infections of the genitourinary tract during pregnancy were significantly associated with functional coding and protein-altering variant models (*p*-values: < 3.60x10^-5^). Sixty-eight of the 71 phenotypes that were marginally significant in the protein-altering variant model were also borderline significant in the functional coding variant analyses (**Supplementary Table 5**).

**Table 3:** Multi-population meta-analysis.

			Rare functional and regulatory model				Functional coding variant model				Protein-altering variant model			
Phecode	Description	Group	P-value	African ancestry cases	European ancestry cases	Total	P-value	African ancestry cases	European ancestry cases	Total	P-value	African ancestry cases	European ancestry cases	Total
170.1	Bone cancer	Neoplasms	0.136904327	75	822	897	9.74049E-05	75	822	897	9.46504E-05	75	822	897
306.9	Tension headache	Mental disorders	**1.78978E-05**	119	902	1021	0.177161406	119	902	1021	0.212721954	119	902	1021
313	Pervasive developmental disorders	Mental disorders	0.011487353	1238	4873	6111	**6.87749E-06**	1238	4873	6111	**6.91666E-06**	1238	4873	6111
313.1	Attention deficit hyperactivity disorder	Mental disorders	0.008254022	1026	3520	4546	0.000114835	1026	3520	4546	0.000118295	1026	3520	4546
402	Elevated blood pressure reading without diagnosis of hypertension	Circulatory system	**2.71696E-05**	206	1140	1346	0.678211569	206	1140	1346	0.726782084	206	1140	1346
535.2	Atrophic gastritis	Digestive	0.083637335	215	909	1124	8.37483E-05	215	909	1124	0.000114423	215	909	1124
588.2	Secondary hyperparathyroidism (of renal origin)	Genitourinary	6.68381E-05	443	1161	1604	0.540609619	443	1161	1604	0.834372718	443	1161	1604
647.1	Infections of genitourinary tract during pregnancy	Pregnancy complications	0.041187233	233	224	457	**1.33627E-05**	234	224	458	**3.55483E-05**	234	224	458
705.1	Dyshidrosis	Dermatologic	7.46523E-05	97	268	365	0.305975243	97	268	365	0.336989681	97	268	365
740.2	Osteoarthrosis, generalized	Musculoskeletal	0.000182261	635	5536	6171	0.636366919	635	5536	6171	0.566502119	635	5536	6171

Bold font represents statistical singificance.

### Sensitivity analyses for pregnancy phenotypes

We performed sensitivity analyses restricted to females with confirmed pregnancies (**Table 4**). In the African ancestry meta-analyses, associations between the three models and 27 pregnancy phenotypes were investigated. Infections of genitourinary tract during pregnancy was significantly associated with every *FOLR2* gene-based model (*p*-values < 2.61x10^-4^). Three other phenotypes had suggestive significance: hemorrhage during pregnancy, childbirth, and the postpartum (*p*-value: 2.35x10^-2^), late pregnancy and failed induction (*p*-value: 4.26x10^-2^), and abnormalities in fetal heart rate or rhythm (*p*-value: 4.58x10^-2^). Thirty-seven pregnancy conditions were evaluated for associations with each of the three gene-based models in the European ancestry meta-analyses. Problems associated with the amniotic cavity and membranes (*p*-value: 2.57x10^-4^) was significantly associated with the rare functional and regulatory variant model. There were no significant relationships detected in the European ancestry functional coding and protein-altering model analyses. However, there were multiple phenotypes with suggestive significance in the European sensitivity analyses, including antepartum hemorrhage, abruptio placentae, and placenta previa, and hypertension complicating pregnancy, childbirth, and the puerperium (**Table 4**). In the multi-ancestry meta-analyses, three of the 27 assessed phenotypes were significantly tied to the rare functional and regulatory variant model: hemorrhage during pregnancy, childbirth, and the postpartum (*p*-value: 5.82x10^-4^), hypertension complicating pregnancy, childbirth, and the puerperium (*p*-value: 1.71x10^-3^), and problems associated with amniotic cavity and membranes (*p*-value: 1.48x10^-5^). Ten additional pregnancy conditions displayed suggestive significance (**Table 4**). Genitourinary tract infections during pregnancy was the only phenotype significantly associated with functional coding and protein-altering models (*p*-values: < 1.22x10^-4^).

**Table 4:** Sensitivity meta-analysis for pregnancy phenotypes.

					Rare functional and regulatory model			Functional coding variant model			Protein-altering variant model		
Phecode	Description	African ancestry cases	European ancestry cases	Multi-population cases	African ancestry P-value	European ancestry P-value	Multi-population P-value	African ancestry P-value	European ancestry P-value	Multi-population P-value	African ancestry P-value	European ancestry P-value	Multi-population P-value
634	Miscarriage;_stillbirth	328	918	1246	0.9986	0.6230	0.4983	0.9115	0.7556	0.8745	0.8062	0.6884	0.7921
634.1	Missed_abortion/Hydatidiform_mole	138	532	670	0.9517	0.1387	0.0551	0.8841	0.9822	0.9825	0.9630	0.9559	0.9951
634.3	Ectopic_pregnancy	54	85	139	0.5621	0.1022	0.0112	0.7718	0.4514	0.5674	0.4629	0.4024	0.4109
635	Hemorrhage_during_pregnancy;_childbirth_and_postpartum	206	555	761	0.0235	0.0112	**5.8208E-04**	0.1802	0.0046	0.0138	0.7952	0.0045	0.0271
635.2	Antepartum_hemorrhage,_abruptio_placentae,_and_placenta_previa	153	421	574	0.0831	0.0312	0.0101	0.0828	0.0090	0.0217	0.5791	0.0091	0.0605
635.3	Placenta_previa_and_abruptio_placenta	58	164	222		0.0111			0.1297			0.1852	
636	Early_or_threatened_labor;_hemorrhage_in_early_pregnancy	1030	2027	3057	0.6077	0.5189	0.3113	0.7009	0.1137	0.3041	0.6343	0.1071	0.2751
636.1	Threatened_premature_labor	338	636	974	0.7903	0.7603	0.5059	0.3972	0.7487	0.6316	0.7397	0.6750	0.8082
636.2	Early_onset_of_delivery	179	516	695	0.4040	0.3728	0.1486	0.5449	0.3315	0.3929	0.6029	0.3271	0.3939
636.3	Hemorrhage_in_early_pregnancy	351	696	1047	0.8404	0.4666	0.2264	0.5977	0.4766	0.7220	0.6144	0.4306	0.7015
636.8	Cervical_incompetence	99	157	256	0.7608	0.2717	0.1193	0.7410	0.4849	0.6657	0.6172	0.8470	0.7538
638	Other_high-risk_pregnancy	154	439	593	0.8682	0.3933	0.4610	0.2415	0.5943	0.4608	0.1960	0.8124	0.4209
642	Hypertension_complicating_pregnancy,_childbirth,_and_the_puerperium	512	965	1477	0.0999	0.0278	**1.7080E-03**	0.3665	0.7567	0.5374	0.3272	0.8522	0.5363
642.1	Preeclampsia_and_eclampsia	227	459	686	0.4156	0.0948	0.0231	0.4360	0.7131	0.6468	0.4153	0.6525	0.6355
643	Excessive_vomiting_in_pregnancy	179	204	383	0.4113	0.3080	0.0363	0.1963	0.3511	0.2165	0.2150	0.3202	0.2348
643.1	Hyperemesis_gravidarum	113	107	220	0.6167	0.1739	0.1591	0.0890	0.9755	0.1495	0.0785	0.9288	0.1371
644	Anemia_during_pregnancy	232	232	464	0.0426	0.0798	0.0024	0.3040	0.9847	0.5816	0.3407	0.9362	0.6324
645	Late_pregnancy_and_failed_induction	33	131	164		0.3257			0.9880			0.9087	
646	Other_complications_of_pregnancy_NEC	473	811	1284	0.4162	0.3437	0.0369	0.1527	0.6027	0.4385	0.2178	0.5886	0.5399
647	Infectious_and_parasitic_complications_affecting_pregnancy	184	220	404	0.2862	0.0546	0.0212	0.0523	1.0000	0.1152	0.1364	0.9984	0.2475
647.1	Infections_of_genitourinary_tract_during_pregnancy	227	224	451	**2.6076E-04**	0.6721	0.0050	**1.8589E-05**	0.6617	**4.3528E-05**	**6.8941E-05**	0.6132	**1.2222E-04**
647.3	Major_puerperal_infection	17	56	73		0.2582			0.2643			0.2578	
649	Other_conditions_or_status_of_the_mother_complicating_pregnancy,_childbirth,_or_the_puerperium	1184	2537	3721	0.5299	0.5302	0.1169	0.7726	0.7939	0.9451	0.7833	0.7626	0.9538
649.1	Diabetes_or_abnormal_glucose_tolerance_complicating_pregnancy	282	786	1068	0.1602	0.4692	0.0589	0.1604	0.5959	0.3446	0.1515	0.5873	0.3353
650	Normal delivery	60	119	179		0.0639			1.0000			0.9999	
651	Multiple gestation	34	70	104		0.4772			0.2890			0.2725	
653	Problems_associated_with_amniotic_cavity_and_membranes	313	845	1158	0.1596	**2.5654E-04**	**1.4836E-05**	0.6441	0.9529	0.9122	0.5752	0.9934	0.8959
654	Other_and_unspecified_complications_of_birth;_puerperium_affecting_management_of_mother	116	411	527	0.7877	0.3288	0.1387	0.6824	0.6730	0.8204	0.7012	0.9637	0.9069
654.1	Abnormality_of_organs_and_soft_tissues_of_pelvis_complicating_pregnancy,_childbirth,_or_the_puerperium	234	282	516	0.7989	0.3806	0.1601	0.3695	0.9980	0.5455	0.2747	0.9851	0.4508
654.2	Rhesus isoimmunization in pregnancy	30	138	168		0.0846			0.0744			0.0996	
655	Known_or_suspected_fetal_abnormality_affecting_management_of_mother	1270	2833	4103	0.3731	0.0668	0.0144	0.8078	0.8207	0.9921	0.7173	0.8113	0.9863
655.1	Abnormality_in_fetal_heart_rate_or_rhythm	610	930	1540	0.0458	0.1318	0.0072	0.3572	0.3575	0.5841	0.2736	0.3480	0.4942
661	Fetal distress and abnormal forces of labor	56	85	141		0.0960			0.1971			0.1867	
665	Obstetrical/birth trauma	120	323	443		0.3583			0.8309			0.7272	
669	Complications_of_labor_and_delivery_NEC	94	344	438	0.9911	0.1199	0.0946	0.4947	0.2178	0.3118	0.6292	0.2072	0.3326
671	Venous/cerebrovascular complications embolism in pregnancy and the puerperium	40	167	207		0.1666			0.9948			0.9520	
674	Other complications of the puerperium NEC	59	116	175		0.6528			0.5674			0.5183	

Bold font represents statistical singificance.

## Discussion

Human genetic studies of *FOLR2* are limited but have identified associations between common variants — including rs13908 and rs651646 — and risk of neural tube defects and congenital heart disease, respectively, with evidence that these effects are modified by maternal folate status (32, 42). Whole-genome sequencing of myelomeningocele cases has further identified ultra-rare deleterious variants in *FOLR2* in a subset of patients (43), but no large-scale GWAS has yet systematically examined *FOLR2* variation in immune or inflammatory disease contexts, representing a significant gap given the receptor’s expression on macrophages and its emerging role in inflammasome biology. However, despite its putative role in folate metabolism, the broader implications of folate in numerous phenotypes, and its specificity to immune cells, the impact of *FOLR2* polymorphisms has not been thoroughly examined. In a 1999 study assessing the impact of *FOLR1* and *FOLR2* on mouse embryo development, Peidrahita and colleagues reported that mice lacking *FOLR1* had severe morphogenetic abnormalities and died *in utero* while mice lacking *FOLR2* developed normally and did not have an obvious phenotype (44).

Here, we systematically evaluated associations between polymorphisms in the *FOLR2* gene and a range of clinical phenotypes. Using PheWAS, we examined the connection between three different *FOLR2* gene-based models (rare functional and regulatory, functional coding, and protein-altering variant gene-based models) and clinical phenotypes using populations from two databases. In ancestry-specific meta-analyses, there were differences in both phenotypes and the number of outcomes that were significantly associated with the three gene-based models. In both ancestry-specific and multi-ancestry meta-analyses, rare functional and regulatory variant models had a larger number of phenotypes that displayed suggestive significance. The results for the functional coding and protein-altering variant models were similar within ancestries. In the multi-population meta-analyses, tension headache was associated with the rare functional and regulatory variant model while pervasive developmental disorders and infections of the genitourinary tract during pregnancy were significantly associated with both the functional coding and protein-altering variant models. Our exploratory association-based study provides initial evidence supporting relationships between these phenotypes and *FOLR2*, though the mechanism underlying these associations are unknown. Below, we summarize results from *in vitro*, animal, and human studies that lend further support to our findings or focus on potential roles of macrophages, folate, FRβ, and/or *FOLR2* in pathogenesis.

In the multi-ancestry meta-analysis, the phecode for pervasive developmental disorders was the top outcome associated with the two models that had the largest impact and consequences on the *FOLR2* gene. The phecode was also significantly associated with the functional coding and protein-altering model in the European ancestry population. In PheWAS coding, pervasive developmental disorders is an umbrella term including attention deficit hyperactivity disorder (ADHD), tics, and autism spectrum disorder (ASD). The bulk of patients with pervasive developmental disorders in our study were diagnosed with ADHD (**Table 2**). Recent meta-analyses have provided evidence that mutations in the folate metabolism gene *MTHFR* are associated with several psychiatric disorders including ADHD and ASD (45, 46).

Though FRβ receptors are primarily present on macrophages, little is known about their role in the cell. In our analysis, several rare variant *FOLR2* gene-based models were tied to phenotypes related to macrophage functions, including conditions with inflammatory and infectious etiology. Notably, we observed a significant connection between *FOLR2* and inflammation of the eye. The association, which was significant in the rare functional and regulatory variant PheWAS in individuals of African ancestry, may be driven by three subsidiary codes: noninfectious conjunctivitis, allergic conjunctivitis, and inflammation of the eyelids. This finding aligns with a previous case report on chronic conjunctivitis in a patient with low folic acid levels and a prospective study demonstrating the potential utility of methotrexate, an antifolate, and folate supplementation for treatment of non-infectious orbital inflammatory disease (47, 48). Alterations in macrophage *FOLR2* receptors due to mutations in the gene may also contribute to this observed relationship, given macrophages role in inducing inflammation.

Dyspareunia, or genital pain around the time of intercourse, was significant or had suggestive significance with multiple *FOLR2* models in several analyses. The link between dyspareunia and *FOLR2* is unclear and complicated by the heterogeneity in etiology of the phenotype, with causes of dyspareunia varying between individuals. However, *FOLR2* may contribute to dyspareunia through several possible mechanisms involving macrophages expression of FRβ. In reproductive age females one of the leading causes of dyspareunia is endometriosis (49), a condition in which macrophages contribute to lesion development and chronic pelvic pain (50). Macrophage activation is also implicated in the pathogenesis of vulvodynia, another leading cause of dyspareunia (51). Dyspareunia is also a frequent occurrence after childbirth, particularly in individuals with complicated deliveries or who experience perineal lacerations (52–54). In these individuals, subsequent pain during intercourse may be due to inflammation, tissue damage, and wound healing. Another possibility is that vaginal pain during intercourse involves macrophages through their roles in the inflammatory response to sexually-transmitted infections (49, 55).

The role of macrophages in defending against infection might explain the observed relationship between *FOLR2* models and infections of the genitourinary tract during pregnancy. This association is interesting given that the tissue exhibiting the highest levels of *FOLR2* expression is the placenta and it is placenta-associated macrophages (both fetal and maternal) that are responsible (16, 20, 56–58). The extent to which FRβ regulates innate immunity during gestation remains to be seen but is an important area warranting future study. In the sensitivity analysis for pregnancy complications, the phecode for hypertension complicating pregnancy, childbirth, and the puerperium was significant in multi-population analysis using the rare functional and regulatory variant model. This phecode includes existing hypertension, transient hypertension of pregnancy, gestational hypertension, preeclampsia, eclampsia, and HELLP (Hemolysis, Elevated Liver enzymes and Low Platelets) syndrome. The subcode combining preeclampsia and eclampsia displayed suggestive significance in the multi-population analysis with the rare functional and regulatory variant model. A recent mouse study using a uterine artery ligation model of preeclampsia demonstrated an accumulation of *FOLR2*^+^ proinflammatory macrophages in the uterus of hypertensive mice (59), which may implicate FRβ in disease pathogenesis.

In recent *in vitro* work, we used CRISPR/Cas9-mediated gene deletion to prevent *FOLR2* expression in the human THP-1 macrophage-like cell line (25). In so doing, we found evidence that *FOLR2* was necessary for optimal caspase-1 activation, gasdermin D cleavage, and IL-1β release in response to multiple stimuli of the NOD-, LRR- and pyrin domain-containing protein 3 (NLRP3) inflammasome (25). These effects were not modified by extracellular folate concentrations and intracellular folic acid concentrations were not influenced by the presence or absence of *FOLR2*. Single-cell RNA sequencing revealed broad transcriptional repression in *FOLR2*-deficient macrophages, including genes involved in inflammasome signaling, and genome-wide methylation profiling showed increased CpG hypermethylation in *FOLR2*-deficient cells, consistent with reduced transcriptional activity. Our *in vitro* data reveals the influence *FOLR2* has on macrophage biology, suggesting that variations in its expression or function could have ramifications *in vivo*.

Our PheWAS approach allowed us to thoroughly investigate potential outcomes of *FOLR2* rare variants. However, results should be taken in context of the limitations of the study, including the data utilized and the PheWAS method. In the present analyses, significance varied based on the population, database, and gene-based model. There are several reasons this variation might be observed. First, there were large differences in sample sizes between the two ancestries, with under 20% of the study populations of predominately African ancestry in both the BioVU and eMERGE. The lower levels of statistical power in the African ancestry analyses may have reduced the probability of detecting a true effect in this population. In addition to failing to detect true relationships between *FOLR2* and phenotypes, sample size imbalance could have further impacted results given that phenotypes were only carried forward to meta-analyses if they had suggestive significance in at least one population and model and had at least 20 cases in each of the populations meta-analyzed. Most statistically significant findings were detected only in meta-analyses, which may suggest that analyses in individual populations were underpowered. Besides power constraints and sample size imbalance, heterogeneity in significant and suggestive results between African and European ancestries could also be due to real differences in genetic architecture between ancestries. Replication of this research using larger sample sizes is needed to verify these results.

While several analyses had small sample sizes, which could have resulted in diminished power to detect true associations, future analyses, with larger sample sizes, could increase power and ensure validity of the results. Results from our pregnancy-specific analyses are compelling but require further validation to ensure our results are not influenced by selection bias, which, though unlikely, could have occurred by excluding individuals whose had been pregnant but did not have records of their pregnancies in the EHRs.

The PheWAS method utilizes phecodes, phenotypes created by aggregating EHR diagnostic and billing codes into broader, clinically relevant groups. This method allowed us to systematically explore the relationship between rare variants in *FOLR2* and disease. The standardized mapping of phecodes allows for easier replications, yet the underlying diagnosis codes may not always match disease status. Appearance of codes could be influenced by social and clinical differences in the diagnosis of disease, coding practice, and insurance. This is particularly relevant for neurodevelopmental and reproductive outcomes. As such, reliance on these codes could lead to bias. To give greater consideration to potential misclassification, we performed analyses using two different EHR-linked DNA biorepositories, one of which combined data from healthcare systems across the U.S. Clinical and billing practices likely differ between the databases and within the eMERGE network. Similar results within the two databases increases our confidence that the possibility of results being due to bias or chance is low. Prospective studies with careful phenotyping should be performed to further refine results and provide estimates of effect.

As both BioVU and eMERGE provided imputed SNP array data, caution should be taken when interpreting results as array-based data could have low imputation quality for rare variants. A likely explanation is due to the models themselves. The rare functional and regulatory variant gene-based model includes all rare nonsynonymous variants, regardless of the impact on the protein. In comparison, the functional coding and protein-altering models are composed of few variants, but all are highly likely or known to impact the encoded protein. This may contribute to the observed phenotypes that were significant in the higher impact models but not in the rare functional and regulatory variant model, like infections of the genitourinary tract during pregnancy. These phenotypes may be driven by significant changes to the FRβ receptor, resulting in changes or complete loss of its function. In contrast, noncoding variants could contribute most to phenotypes which were only significant in rare functional and regulatory model analyses. These variants likely have functional impact outside of protein disruption (e.g., regulatory control). Computational or *in vitro* approaches that focus on FRβ’s ability to bind to folic acid and facilitate its delivery to the interior of cells would also add further strength and clarity to our results.

Results from analyses using whole genome or whole exome sequencing data would have more strength. However, our analysis is an important initial step in this research, given the lack of existing research on *FOLR2*. Our findings are also strengthened by using multiple databases, ancestries, and gene-based model classification, as well as their alignment with current knowledge, results from animal models, and/or biologic mechanisms. We also utilized a stringent significance threshold (Bonferroni correction) to further reduce the probability of false associations.

Though we investigated genetic mutations in *FOLR2*, it is important to further contextualize these findings. Folate metabolism is influenced by diet and supplementation, aspects not always reliably or accurately captured in EHRs. These environmental exposures may modify associations between folate related genes and outcomes. Computational and *in vivo* approaches investigating how rare variants change the FRβ receptor and overall folate metabolism are first necessary. Then population-based studies could be used to deduce whether diet and folate supplementation can amplify or lessen the impact these mutations have on folate metabolism and resulting outcomes.

Research on polymorphisms in the *FOLR2* gene and their functional consequences is scarce. Existing research largely focuses on other folate-related genes or limits investigated outcomes to neural tube defects or cancer. Combining SKAT-O gene-based models and PheWAS approach, we furthered research on *FOLR2* by systematically evaluating associations between rare variants in the *FOLR2* gene and clinical phenotypes. We found significant associations between models and neurodevelopmental and infectious disease phenotypes. Interestingly, we also detected significant associations between *FOLR2* and phenotypes related to macrophage function, including inflammation of the eye, genitourinary tract infections in pregnancy, and dyspareunia. These associations require further investigation given the presence of *FOLR2* receptors on macrophages but their unknown role for the immune cells.

## Data Availability

The data analyzed in this study is subject to the following licenses/restrictions: Individual-level data for this manuscript cannot be made readily available due to institutional restrictions but is readily available through IRB and proposal approval to Vanderbilt University Medical Center or the Electronic Medical Records and Genomics network. Full results for all analyses are accessible and provided in **Supplementary Tables**. Requests to access these datasets should be directed to Digna Velez-Edwards digna.r.velez.edwards@vumc.org.

## References

[1] Yi YS . Folate receptor-targeted diagnostics and therapeutics for inflammatory diseases. Immune Netw. (2016) 16:337–43. doi: 10.4110/in.2016.16.6.337 28035209 PMC5195843

[2] Menezo Y Elder K Clement A Clement P . Folic acid, folinic acid, 5 methyl tetrahydrofolate supplementation for mutations that affect epigenesis through the folate and one-carbon cycles. Biomolecules. (2022) 12(2):197. doi: 10.3390/biom12020197 35204698 PMC8961567

[3] Laurence KM James N Miller MH Tennant GB Campbell H . Double-blind randomised controlled trial of folate treatment before conception to prevent recurrence of neural-tube defects. Br Med J (Clin Res Ed). (1981) 282:1509–11. doi: 10.1097/00006254-198111000-00013 6786536 PMC1505459

[4] Santander Ballestin S Gimenez Campos MI Ballestin Ballestin J Luesma Bartolome MJ . Is supplementation with micronutrients still necessary during pregnancy? A review. Nutrients. (2021) 13:3134. doi: 10.3390/nu13093134 34579011 PMC8469293

[5] Gok V Erdem S Haliloglu Y Bisgin A Belkaya S Basaran KE . Immunodeficiency associated with a novel functionally defective variant of SLC19A1 benefits from folinic acid treatment. Genes Immun. (2023) 24:12–20. doi: 10.1038/s41435-022-00191-7 36517554

[6] Matherly LH Schneider M Gangjee A Hou Z . Biology and therapeutic applications of the proton-coupled folate transporter. Expert Opin Drug Metab Toxicol. (2022) 18:695–706. doi: 10.1080/17425255.2022.2136071 36239195 PMC9637735

[7] Chandrupatla D Molthoff CFM Lammertsma AA Van Der Laken CJ Jansen G . The folate receptor beta as a macrophage-mediated imaging and therapeutic target in rheumatoid arthritis. Drug Delivery Transl Res. (2019) 9:366–78. doi: 10.1007/s13346-018-0589-2 30280318 PMC6328514

[8] Sabharanjak S Mayor S . Folate receptor endocytosis and trafficking. Adv Drug Delivery Rev. (2004) 56:1099–109. doi: 10.1016/j.addr.2004.01.010 15094209

[9] Varghese B Vlashi E Xia W Ayala Lopez W Paulos CM Reddy J . Folate receptor-beta in activated macrophages: ligand binding and receptor recycling kinetics. Mol Pharm. (2014) 11:3609–16. doi: 10.1021/mp500348e 25166491

[10] Matherly LH Diop-Bove N Goldman ID . Biological role, properties, and therapeutic applications of the reduced folate carrier (RFC-SLC19A1) and the proton-coupled folate transporter (PCFT-SLC46A1). In: Jackman AL Leamon CP , editors. Targeted Drug Strategies for Cancer and Inflammation. Springer US, Boston, MA (2011). p. 1–34.

[11] Bianchi E Doe B Goulding D Wright GJ . Juno is the egg Izumo receptor and is essential for mammalian fertilization. Nature. (2014) 508:483–7. doi: 10.1038/nature13203 24739963 PMC3998876

[12] Holm J Hansen SI . Characterization of soluble folate receptors (folate binding proteins) in humans. Biological roles and clinical potentials in infection and Malignancy. Biochim Biophys Acta Proteins Proteom. (2020) 1868:140466. doi: 10.1016/j.bbapap.2020.140466 32526472

[13] Otsubo H Tsuneyoshi Y Nakamura T Matsuda T Komiya S Matsuyama T . Serum-soluble folate receptor beta as a biomarker for the activity of rheumatoid arthritis synovitis and the response to anti-TNF agents. Clin Rheumatol. (2018) 37:2939–45. doi: 10.1007/s10067-018-4202-3 30022370

[14] Ross JF Chaudhuri PK Ratnam M . Differential regulation of folate receptor isoforms in normal and Malignant tissues *in vivo* and in established cell lines. Physiologic and clinical implications. Cancer. (1994) 73:2432–43. doi: 10.1002/1097-0142(19940501)73:9<2432::aid-cncr2820730929>3.0.co;2-s 7513252

[15] Feng Y Shen J Streaker ED Lockwood M Zhu Z Low PS . A folate receptor beta-specific human monoclonal antibody recognizes activated macrophage of rheumatoid patients and mediates antibody-dependent cell-mediated cytotoxicity. Arthritis Res Ther. (2011) 13:R59. doi: 10.1186/ar3312 21477314 PMC3132054

[16] Digre A Lindskog C . The human protein atlas-integrated omics for single cell mapping of the human proteome. Protein Sci. (2023) 32:e4562. doi: 10.1002/pro.4562 36604173 PMC9850435

[17] Svensson J Jenmalm MC Matussek A Geffers R Berg G Ernerudh J . Macrophages at the fetal-maternal interface express markers of alternative activation and are induced by M-CSF and IL-10. J Immunol. (2011) 187:3671–82. doi: 10.4049/jimmunol.1100130 21890660

[18] Tang Z Buhimschi IA Buhimschi CS Tadesse S Norwitz E Niven-Fairchild T . Decreased levels of folate receptor-beta and reduced numbers of fetal macrophages (Hofbauer cells) in placentas from pregnancies with severe pre-eclampsia. Am J Reprod Immunol. (2013) 70:104–15. doi: 10.1111/aji.12112 23480364 PMC3686834

[19] Vento-Tormo R Efremova M Botting RA Turco MY Vento-Tormo M Meyer KB . Single-cell reconstruction of the early maternal-fetal interface in humans. Nature. (2018) 563:347–53. doi: 10.1038/s41586-018-0698-6 30429548 PMC7612850

[20] Thomas JR Appios A Zhao X Dutkiewicz R Donde M Lee CYC . Phenotypic and functional characterization of first-trimester human placental macrophages, Hofbauer cells. J Exp Med. (2021) 218:e20200891. doi: 10.1084/jem.20200891 33075123 PMC7579740

[21] Sureshchandra S Doratt BM True H Mendoza N Rincon M Marshall NE . Multimodal profiling of term human decidua demonstrates immune adaptations with pregravid obesity. Cell Rep. (2023) 42:112769. doi: 10.1016/j.celrep.2023.112769 37432849 PMC10528932

[22] Branco A Rogers LM Aronoff DM . Folate receptor beta signaling in the regulation of macrophage antimicrobial immune response: A scoping review. BioMed Hub. (2024) 9:31–7. doi: 10.1159/000536186 38406385 PMC10890800

[23] Samaniego R Palacios BS Domiguez-Soto A Vidal C Salas A Matsuyama T . Macrophage uptake and accumulation of folates are polarization-dependent *in vitro* and *in vivo* and are regulated by activin A. J Leukoc Biol. (2014) 95:797–808. doi: 10.1189/jlb.0613345 24399840

[24] Ritchie C Cordova AF Hess GT Bassik MC Li L . SLC19A1 is an importer of the immunotransmitter cGAMP. Mol Cell. (2019) 75:372–81. doi: 10.1016/j.molcel.2019.05.006 31126740 PMC6711396

[25] Rogers LM Firestone K Chinni R Branco AC Wrobleski K Chou T . Folate receptor beta drives NLRP3 inflammasome activation and pyroptosis in macrophages independent of folate binding. J Immunol. (2026) 215:1–25. doi: 10.1093/jimmun/vkag051 41984502 PMC13351659

[26] Winkler CW Evans AB Carmody AB Peterson KE . Placental Myeloid Cells Protect against Zika Virus Vertical Transmission in a Rag1-Deficient Mouse Model. J Immunol. (2020) 205:143–52. doi: 10.4049/jimmunol.204.supp.248.14 PMC8328348 32493813

[27] Gay L Madariaga Zarza S Abou Atmeh P Rouviere MS Andrieu J Richaud M . Protective role of macrophages from maternal-fetal interface in unvaccinated coronavirus disease 2019 pregnant women. J Med Virol. (2024) 96:e29819. doi: 10.1002/jmv.29819 39030992

[28] Schuch V Hossack D Hailstorks T Chakraborty R Johnson EL . Distinct immune responses to HIV and CMV in Hofbauer cells across gestation highlight evolving placental immune dynamics. Front Immunol. (2026) 17:832988. doi: 10.3389/fimmu.2026.1832988 PMC13286909 42344922

[29] Klerk M Verhoef P Clarke R Blom HJ Kok FJ Schouten EG . MTHFR 677C-->T polymorphism and risk of coronary heart disease: a meta-analysis. JAMA. (2002) 288:2023–31. 10.1001/jama.288.16.2023 12387655

[30] Kennedy DA Stern SJ Matok I Moretti ME Sarkar M Adams-Webber T . Folate intake, MTHFR polymorphisms, and the risk of colorectal cancer: A systematic review and meta-analysis. J Cancer Epidemiol. (2012) 2012:952508. doi: 10.1155/2012/952508 23125859 PMC3483802

[31] Yan L Zhao L Long Y Zou P Ji G Gu A . Association of the maternal MTHFR C677T polymorphism with susceptibility to neural tube defects in offsprings: evidence from 25 case-control studies. PloS One. (2012) 7:e41689. doi: 10.1371/journal.pone.0041689 23056169 PMC3463537

[32] Song X Wei J Shu J Liu Y Sun M Zhu P . Association of polymorphisms of FOLR1 gene and FOLR2 gene and maternal folic acid supplementation with risk of ventricular septal defect: a case-control study. Eur J Clin Nutr. (2022) 76:1273–80. doi: 10.1038/s41430-022-01110-9 35273364

[33] Denny JC Ritchie MD Basford MA Pulley JM Bastarache L Brown-Gentry K . PheWAS: demonstrating the feasibility of a phenome-wide scan to discover gene-disease associations. Bioinformatics. (2010) 26:1205–10. doi: 10.1093/bioinformatics/btq126 20335276 PMC2859132

[34] Roden DM Pulley JM Basford MA Bernard GR Clayton EW Balser JR . Development of a large-scale de-identified DNA biobank to enable personalized medicine. Clin Pharmacol Ther. (2008) 84:362–9. doi: 10.1038/clpt.2008.89 18500243 PMC3763939

[35] Gottesman O Kuivaniemi H Tromp G Faucett WA Li R Manolio TA . The Electronic Medical Records and Genomics (eMERGE) Network: past, present, and future. Genet Med. (2013) 15:761–71. doi: 10.1038/gim.2013.72 23743551 PMC3795928

[36] Das S Forer L Schonherr S Sidore C Locke AE Kwong A . Next-generation genotype imputation service and methods. Nat Genet. (2016) 48:1284–7. doi: 10.1038/ng.3656 27571263 PMC5157836

[37] Taliun D Harris DN Kessler MD Carlson J Szpiech ZA Torres R . Sequencing of 53,831 diverse genomes from the NHLBI TOPMed Program. Nature. (2021) 590:290–9. doi: 10.1038/s41586-021-03205-y 33568819 PMC7875770

[38] Zuvich RL Armstrong LL Bielinski SJ Bradford Y Carlson CS Crawford DC . Pitfalls of merging GWAS data: lessons learned in the eMERGE network and quality control procedures to maintain high data quality. Genet Epidemiol. (2011) 35:887–98. doi: 10.1002/gepi.20639 22125226 PMC3592376

[39] Mccarthy S Das S Kretzschmar W Delaneau O Wood AR Teumer A . A reference panel of 64,976 haplotypes for genotype imputation. Nat Genet. (2016) 48:1279–83. doi: 10.1101/035170 PMC5388176 27548312

[40] Harrison PW Amode MR Austine-Orimoloye O Azov AG Barba M Barnes I . Ensembl 2024. Nucleic Acids Res. (2024) 52:D891–9. doi: 10.1093/nar/gkad1049 37953337 PMC10767893

[41] Lee S Emond MJ Bamshad MJ Barnes KC Rieder MJ Nickerson DA . Optimal unified approach for rare-variant association testing with application to small-sample case-control whole-exome sequencing studies. Am J Hum Genet. (2012) 91:224–37. doi: 10.1016/j.ajhg.2012.06.007 22863193 PMC3415556

[42] O'byrne MR Au KS Morrison AC Lin JI Fletcher JM Ostermaier KK . Association of folate receptor (FOLR1, FOLR2, FOLR3) and reduced folate carrier (SLC19A1) genes with meningomyelocele. Birth Defects Res A Clin Mol Teratol. (2010) 88:689–94. doi: 10.1002/bdra.20706 PMC3046546 20683905

[43] Au KS Hebert L Hillman P Baker C Brown MR Kim DK . Human myelomeningocele risk and ultra-rare deleterious variants in genes associated with cilium, WNT-signaling, ECM, cytoskeleton and cell migration. Sci Rep. (2021) 11:3639. doi: 10.1038/s41598-021-83058-7 33574475 PMC7878900

[44] Piedrahita JA Oetama B Bennett GD Van Waes J Kamen BA Richardson J . Mice lacking the folic acid-binding protein Folbp1 are defective in early embryonic development. Nat Genet. (1999) 23:228–32. doi: 10.1038/13861 10508523

[45] Li Y Qiu S Shi J Guo Y Li Z Cheng Y . Association between MTHFR C677T/A1298C and susceptibility to autism spectrum disorders: a meta-analysis. BMC Pediatr. (2020) 20:449. doi: 10.1186/s12887-020-02330-3 32972375 PMC7517654

[46] Meng X Zheng JL Sun ML Lai HY Wang BJ Yao J . Association between MTHFR (677C>T and 1298A>C) polymorphisms and psychiatric disorder: A meta-analysis. PloS One. (2022) 17:e0271170. doi: 10.1371/journal.pone.0271170 35834596 PMC9282595

[47] Smith JR Rosenbaum JT . A role for methotrexate in the management of non-infectious orbital inflammatory disease. Br J Ophthalmol. (2001) 85:1220–4. doi: 10.1136/bjo.85.10.1220 11567968 PMC1723732

[48] Malm E Ghosh F . Chronic conjunctivitis in a patient with folic acid deficiency. Acta Ophthalmol Scand. (2007) 85:226. doi: 10.1111/j.1600-0420.2006.00801.x 17305744

[49] Carlson K Mikes BA . Dyspareunia. In: Statpearls. StatPearls Publishing LLC, Treasure Island (FL (2026). 32965830

[50] Nati ID Ciortea R Malutan A Oancea M Iuhas C Bucuri C . Dyspareunia and biomarkers: A case study of sexual dysfunction in moderate endometriosis. Int J Mol Sci. (2024) 26. doi: 10.3390/ijms26010162 39796020 PMC11720033

[51] Barry CM Matusica D Haberberger RV . Emerging evidence of macrophage contribution to hyperinnervation and nociceptor sensitization in vulvodynia. Front Mol Neurosci. (2019) 12:186. doi: 10.3389/fnmol.2019.00186 31447644 PMC6691023

[52] Manresa M Pereda A Bataller E Terre-Rull C Ismail KM Webb SS . Incidence of perineal pain and dyspareunia following spontaneous vaginal birth: a systematic review and meta-analysis. Int Urogynecol J. (2019) 30:853–68. doi: 10.1007/s00192-019-03894-0 30770967

[53] Cattani L De Maeyer L Verbakel JY Bosteels J Deprest J . Predictors for sexual dysfunction in the first year postpartum: A systematic review and meta-analysis. BJOG. (2022) 129:1017–28. doi: 10.22541/au.161330612.22257589/v1 34536325

[54] Josefsson ML Sohlberg S Ekeus C Uustal E Jonsson M . Self-reported dyspareunia and outcome satisfaction after spontaneous second-degree tear compared to episiotomy: A register-based cohort study. PloS One. (2024) 19:e0315899. doi: 10.1371/journal.pone.0315899 39700239 PMC11658579

[55] Iijima N Thompson JM Iwasaki A . Dendritic cells and macrophages in the genitourinary tract. Mucosal Immunol. (2008) 1:451–9. doi: 10.1038/mi.2008.57 19079212 PMC2684461

[56] Iijima N Thompson JM Iwasaki A . Dendritic cells and macrophages in the genitourinary tract.. Mucosal Immunol. (2008) 1(6):451–9. doi: 10.1038/mi.2008.57 PMC2684461 19079212

[57] Uhlen M Karlsson MJ Zhong W Tebani A Pou C Mikes J . A genome-wide transcriptomic analysis of protein-coding genes in human blood cells. Science. (2019) 366(6472):eaax9198. doi: 10.1126/science.aax9198 31857451

[58] Karlsson M Zhang C Mear L Zhong W Digre A Katona B . A single-cell type transcriptomics map of human tissues. Sci Adv. (2021) 7(31):eabh2169. doi: 10.1126/sciadv.abh2169 34321199 PMC8318366

[59] Fei H Lu X Shi Z Liu X Yang C Zhu X . Deciphering the preeclampsia-specific immune microenvironment and the role of pro-inflammatory macrophages at the maternal-fetal interface. Elife. (2025) 13:RP100002. doi: 10.7554/elife.100002.3 PMC11952753 40152904

